# CH_4_, C_2_H_6_, and CO_2_ Multi-Gas Sensing Based on Portable Mid-Infrared Spectroscopy and PCA-BP Algorithm

**DOI:** 10.3390/s23031413

**Published:** 2023-01-27

**Authors:** Yunting Yang, Jiachen Jiang, Jiafu Zeng, Zhangxiong Chen, Xiaosong Zhu, Yiwei Shi

**Affiliations:** 1 Key Laboratory for Information Science of Electromagnetic Waves (MoE), Fudan University, Shanghai 200433, China; 2 Zhongshan–Fudan Joint Innovation Center, Zhongshan 528437, China

**Keywords:** multi-gas detection, NDIR, PCA, BP neural network

## Abstract

A multi-gas sensing system was developed based on the detection principle of the non-dispersive infrared (NDIR) method, which used a broad-spectra light source, a tunable Fabry–Pérot (FP) filter detector, and a flexible low-loss infrared waveguide as an absorption cell. CH_4_, C_2_H_6_, and CO_2_ gases were detected by the system. The concentration of CO_2_ could be detected directly, and the concentrations of CH_4_ and C_2_H_6_ were detected using a PCA-BP neural network algorithm because of the interference of CH_4_ and C_2_H_6_. The detection limits were achieved to be 2.59 ppm, 926 ppb, and 114 ppb for CH_4_, C_2_H_6_, and CO_2_ with an averaging time of 429 s, 462 s, and 297 s, respectively. The root mean square error of prediction (RMSEP) of CH_4_ and C_2_H_6_ were 10.97 ppm and 2.00 ppm, respectively. The proposed system and method take full advantage of the multi-component gas measurement capability of the mid-infrared broadband source and achieve a compromise between performance and system cost.

## 1. Introduction

Multi-gas detection plays an important role in many areas, such as medical diagnosis, industrial application, environmental atmospheric monitoring, and fire alarm systems in coal mines [1,2,3]. So far, the most widely used techniques in infrared spectroscopy for multi-gas detection are the tunable diode laser absorption spectroscopy (TDLAS) and non-dispersive infrared (NDIR) detection system.

TDLAS has the advantages of high precision, high sensitivity, and good selectivity. Zou et al. reported a near-infrared dual-gas sensing system for methane (CH_4_) and ethane (C_2_H_6_) based on a distributed feedback (DFB) diode laser in the near-infrared region, and the detection limits were about 23.53 ppb for CH_4_ and 146.4 ppb for C_2_H_6_ in 200 s [4]. However, the cost of TDLAS is high and its tunable spectral range is narrow, which means that it can only be used for one type of gas or multiple gases with adjacent absorption lines. Multiple lasers are necessary if TDLAS is used for multi-gas detection in a wide spectral range, which caused system complexity and increased costs. Xi et al. developed a near-infrared dual-gas sensor system for CH_4_ and C_2_H_6_ using two DFB diode lasers, and the detection limits were about 78 ppb for CH_4_ and 190 ppb for C_2_H_6_ in 0.8 s [5]. Piotr Jaworski et al. realized a dual-gas sensor for the detection of carbon dioxide (CO_2_) and CH_4_ in the near- and mid-infrared regions using a DFB diode laser and a custom-made MIR laser based on a difference frequency generation phenomenon, and it reached a detection limit down to 24 ppb for CH_4_ and 144 ppm for CO_2_ [6]. 

NDIR has the advantages of a simple system, low cost, wide detection range, and moderate sensitivity. Hence, it is widely used for multi-gas detection, and has been used to measure the concentration of more than 100 types of gases. However, NDIR has the disadvantage of a low resolution, so there is always the problem of interference in multi-gas detection. To solve the problem, optical filters and the concentration inversion models are always applied in the NDIR. Xu et al. developed a NDIR multi-gas detection system consisting of a single broadband light source and four-channel pyroelectric detector to analyse CO_2_, carbon monoxide (CO), and propane (C_3_H_8_), and it was observed that the full-scale error of the sensor changed less than 3.5%, the detection repeatability error was lower than 4.5%, and the detection stability was less than 2.7% [7]. Liu et al. proposed a NDIR-system-based four-channel thermoelectricity detector to analyse CO and CO_2_, and the detector’s data processor has 3% accuracy and stability [8]. Dong et al. developed a multi-gas sensor system that used a single broadband light source and three pyroelectric detectors by use of the time division multiplexing (TDM) technique, and the detection limits were about 2.96, 4.54, and 2.84 ppm for CO, CO_2_, and CH_4_, respectively [9]. 

In this paper, CH_4_, C_2_H_6_, CO_2_, and their mixtures were detected. CO_2_ is the most abundant greenhouse gas. CH_4_ is one of the most important greenhouse gases, contributing 25 times more to global warming than CO_2_ in 100 years [10,11,12]. C_2_H_6_ is another important greenhouse gas that damages the ozone layer [13,14]. In addition to CH_4_ and C_2_H_6_ being two characteristic gases for monitoring transformer status in dissolved gas analysis (DGA), they are also the first and second largest components of natural gas [15,16,17].

A miniaturized NDIR sensing system was established. A blackbody radiation broad-spectra light source and a tunable Fabry–Pérot (FP) filter detector were used [18,19]. The detection wavelength range can be controlled by adjusting the driving voltage of the FP interferometer. A homemade flexible waveguide was used as the gas absorption cell which could improve the portability of the system [20,21]. The sensing system attained high performances because of the long optical path and low loss of the flexible waveguide. Multi-gas detection always has the problem of interference in the spectrum. Intelligent learning methods can solve this problem and have achieved good results [22,23,24,25,26]. Hence, we used principal component analysis (PCA) and the back propagation (BP) neural network to correct the interference of multi-gas detection, and improved the detection performance of the system. A simulation-aid training method is also proposed in this paper to reduce the time cost. In both hardware and software considerations, the system proposed in this paper achieves a compromise between performances and system cost.

## 2. Principle and System 

### 2.1. Sensing System Design

**Figure 1:**
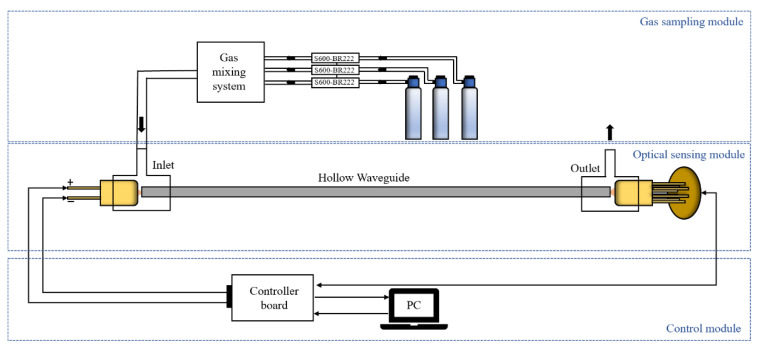
Schematic diagram of the sensing system.

**Figure 2:**
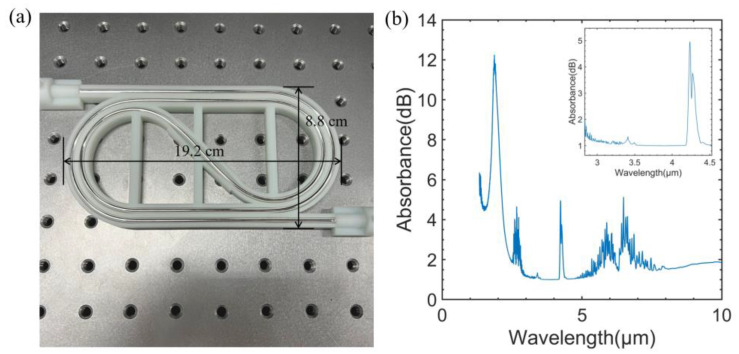
(**a**) Photo of the flexible HWG with 100 cm length. (**b**) Measured loss spectra of the HWG with 100 cm length.

The schematic diagram of the sensing system is shown in Figure 1, which is generally divided into three modules: gas dilution module, optical sensing module, and control module. In the optical sensing module, a broadband thermal light source (Axetris, EMIRS200, Sarnen, Switzerland) was chosen as the infrared light source. Its emission spectrum range is 2~14 μm, which covers the absorption band of CH_4_ (3.2~3.6 μm), C_2_H_6_ (3.2~3.6 μm), and CO_2_ (4.2~4.4 μm). A tunable Fabry–Pérot filter detector (Infratech, LFP-3144(C)-337, Dresden, Germany) was used as the detector with a tuning wavelength range from 3.1 to 4.4 µm. The wavelength resolution of the FP detector is low, which is about 60 nm. The hollow waveguide (HWG) can simultaneously serve as a transmission medium and gas absorption cell for mid-infrared gas sensing. It has the advantages of low loss, small volume, flexibility, and fast response [27]. A polycarbonate base tube was chosen for HWG to achieve flexibility. A silver iodide and silver (AgI/Ag) were inner-coated to achieve low loss for the HWG at the target wavelength. Figure 2a shows that the HWG has good flexibility and can be bent into the small size substrate, which improved the portability of the system. The length and inner diameter of the HWG applied in this work are 100 cm and 3.5 mm, respectively. Figure 2b shows the measured loss spectrum of the 100 cm length AgI/Ag waveguide by the FTIR. Low-loss property in the wavelength band from 3.1 to 4.4 µm was attained. The peak around 4.3 µm is the absorption of CO_2_ in the air. The HWG was directly connected with the light source and detector by 3D printed waveguide splices without any focal lenses. The control module was mainly composed of the personal computer and the controller board (Infratech, FPI-EvalKit, Dresden, Germany). The controller board was connected through the computer to set the driving current of the light source and the measurement step of the FP filter detector, and receive the signal detected by the detector. The measurement wavelength step set in this work was 20 nm and 66 data points were collected over the wavelength range of 1300 nm. The time scanning across the whole spectrum was about 33 s. The gas dilution module was composed of three mass flow controllers (HORIBA, S600-BR222, Shanghai, China, with a 1% uncertainty) and a gas mixing pipe. The flow rate of each mass flow controller was set by computer to get different concentrations of mixture gas. The standard gases used in this work were high-purity nitrogen (N_2_ ≥ 99.999%, H_2_O ≤ 3 ppm, CO_2_ ≤ 1 ppm, Chemical Center of Fudan University, Shanghai, China), standard 3040 ppm CH_4_ gas (Air Liquid, Shanghai, China), standard 311 ppm CH_4_ gas (Air Liquid, Shanghai, China), standard 3040 ppm C_2_H_6_ gas (Air Liquid, Shanghai, China), and standard 100 ppm CO_2_ gas (Air Liquid, Shanghai, China).

### 2.2. Principle

The basic principle of the gas sensor is the Beer–Lambert law:

(1) $$A\left(v\right)=ln\frac{I_{0}\left(v\right)}{I_{t}\left(v\right)}=K\left(v\right)\cdot L\cdot C$$

where *v* is the frequency of incident infrared light (cm^−1^), *A* is the absorbance (dB), *I*_0_ is the intensity of incident light, *I*_𝑡_ is the intensity of transmitted light, *K* is the absorption cross-section (cm^2^/molecule), *L* is the optical path length (cm), and *C* is the concentration of gas (molecule/cm^3^).

**Figure 3:**
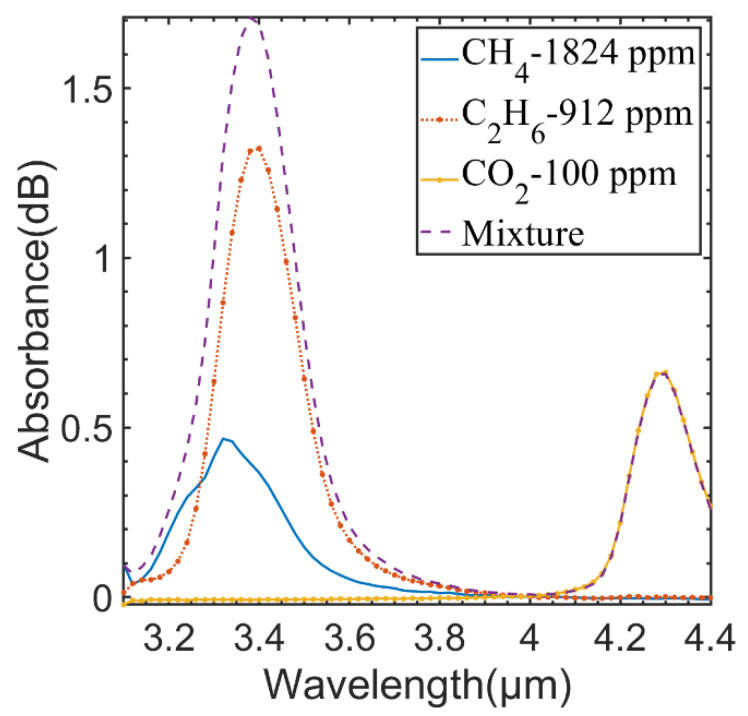
Measured absorption spectra of CH_4_, C_2_H_6_, and CO_2_ by the FPI sensing system.

Figure 3 shows the absorption spectra of three single-gas samples and their mixture sample measured by the established sensing system. CH_4_ and C_2_H_6_ have great interference in the wavelength range of 3.2~3.6 μm, and CO_2_ has little interference with the other two gases. Hence, CO_2_ can be detected directly around the 4.3 μm wavelength. The absorption spectra of CH_4_ and C_2_H_6_ are highly overlapping and cannot be detected directly, so a nonlinear fitting algorithm must be used to correct the interference of CH_4_ and C_2_H_6_. In this paper, the PCA-BP neural network algorithm was used to obtain the concentrations of the two gases from the interference mixed gas absorption spectra. 

According to the Beer–Lambert law, when the scattering and the influence of the system are not considered, the absorbance *A* is proportional to the concentration *C*. Therefore, the concentration *C* can be calculated from the absorption spectra directly. In the interference mixed gas spectra, the absorbance *A* of the spectra is not linear to the concentrations of CH_4_ and C_2_H_6_. This is because the components of the mixture sample have the interaction and the instrument has background noise. The BP neural network can approximate some nonlinear relation functions well, so it was applied to obtain the relationship between absorbance *A* and concentrations of CH_4_ and C_2_H_6_. However, the number of data points for absorption *A* is too large, which leads to the long training time. Therefore, PCA was used to reduce the dimensions of absorbance *A*.

PCA is a dimensionality reduction method, which aims to replace many variables with fewer variables and can reflect most of the information of the original many variables.

For the sample X_n×p_ with *p* variables and n data, the covariance matrix Σ_p×p_ can be calculated. According to the covariance matrix, *p* eigenvalues can be calculated and sorted from large to small *λ*_1_, *λ*_2_… *λ*_P_, *p* eigenvectors can also be calculated and sorted from large to small *T*_1_, *T*_2_… *T*_p_. Then, the *i*th principal component *Y_i_* is as follows:

(2) $$Y_{i}=X_{i}T_{i},\;1\leq i\leq p$$

There are *p* variables in the original sample, and the number of variables will be greatly reduced after principal component analysis. The number of principal components shall be selected according to the principal component contribution rate and cumulative contribution rate. The contribution rate of the *k*th principal component is as follows:

(3) $$e_{k}=\frac{\lambda_{k}}{\sum_{i=1}^{p}\lambda_{i}}$$

Generally, the greater the contribution rate of the principal component, the more information about the original data is saved. The cumulative contribution rate of the first *m* principal components of the sample is as follows:

(4) $$E_{m}=\frac{\sum_{i=1}^{m}\lambda_{i}}{\sum_{i=1}^{p}\lambda_{i}}=\sum_{k=1}^{m}e_{k}$$

The cumulative contribution rate is the standard to judge the number of selected principal components, and also reflects the retention of original information by these principal components.

**Figure 4:**
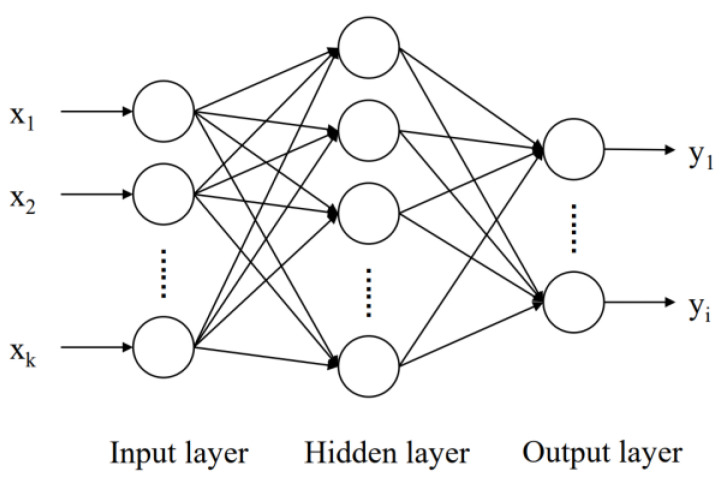
Topology of BP neural network.

BP neural network is a neural network model trained by error back propagation. It can realize any nonlinear mapping, so it is very suitable for solving the nonlinear absorption effect of multi-gas. BP neural network includes input layer, hidden layer, and output layer, as shown in Figure 4. The calculation process of the BP neural network is forward, from the input layer to the hidden layer and then to the output layer. If the results of the output layer cannot reach the expected value, then error calculation and parameter correction will be carried out. This step is performed through reverse propagation to minimize the error of the output results, so as to obtain the trained BP neural network model.

Let input *X* have *k* variables *x*_1_, *x*_2_… *x_k_*, so the number of input layer nodes is *k*. Let the weight matrix be *W*, and the offset value be *B*. The nonlinear mapping is realized through the excitation function. The excitation function used in this paper is the sigmoid function, as shown in Equation (5):

(5) $$f\left(x\right)=\frac{e^{x}-e^{-x}}{e^{x}+e^{-x}}$$

The output value O of the neural network node is shown in Equation (6):

(6) O=f(X∗W+B)

In the process of error back propagation, the Levenberg–Marquardt algorithm is used to update the weight matrix and offset value of the hidden layer and output layer, so as to achieve the trained BP neural network model.

## 3. Results and Discussion

### 3.1. Performance of the Sensor for Single Gas

**Figure 5:**
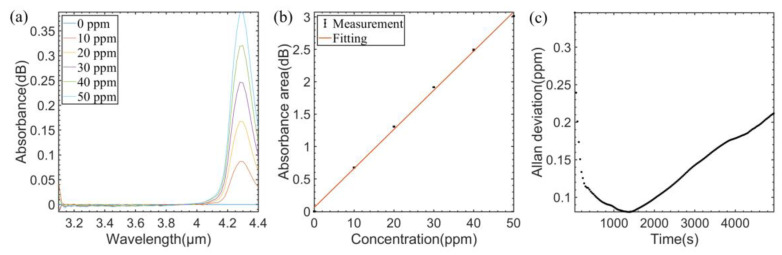
(**a**) Measured absorption spectra of different CO_2_ concentrations. (**b**) Experimental data and fitting curve of CO_2_ concentration versus absorbance area. (**c**) Allan variance analysis of the sensor for CO_2_.

The performance of the system for single gas detection was evaluated by introducing three single gases at different concentrations into the system, respectively. As shown in Figure 5a, the concentration of CO_2_ varied from 0 ppm to 50 ppm using the gas dilution module. The absorption peak areas over the spectral range of 4.2~4.4 µm were recorded. Each absorption spectrum was measured five times and the average value of the absorption spectrum was used. The absorbance area is linear to the CO_2_ concentrations, as shown in Figure 5b. The linear relationship is expressed by Equation (7) with the R square value of 0.9982, as follows:

(7) $$A_{CO_{2}}=0.0604*c_{CO_{2}}+0.0586$$

with *A* and *c* denoting the CO_2_ absorbance area and the CO_2_ concentration, respectively.

Then, the CO_2_ sample with a concentration of 0 ppm was injected into the HWG to observe the stability of the whole system. 317 sets of data were collected in 3 h. Allan variance analysis was applied to evaluate the detection limit of the system, as shown in Figure 5c. The Allan deviation for CO_2_ detection is 114 ppb at an averaging time of 297 s.

**Figure 6:**
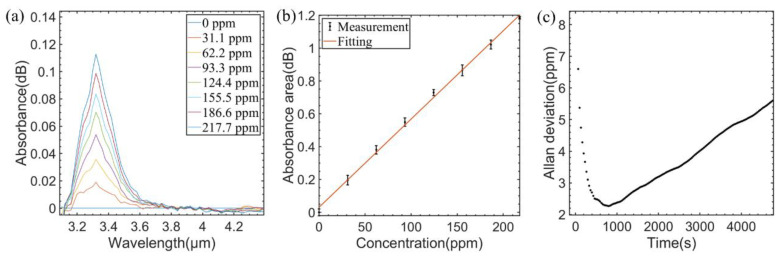
(**a**) Measured absorption spectra of different CH_4_ concentrations. (**b**) Experimental data and fitting curve of CH_4_ concentration versus absorbance area. (**c**) Allan variance analysis of the sensor for CH_4_.

Using the same experimental approach, the absorption spectra measured for different concentrations of CH_4_ (with CH_4_ concentration varying from 0 ppm to 217.7 ppm, the variation interval was 31.1 ppm) are shown in Figure 6a. Figure 6b shows the linear fitting between absorbance area and CH_4_ concentrations. The R square value is 0.9976 and the fitting function is expressed as follows:

(8) $$A_{CH_{4}}=0.0054*c_{CH_{4}}+0.0303$$

As shown in Figure 6c, the Allan deviation for CH_4_ detection is 2.59 ppm at an averaging time of 429 s.

**Figure 7:**
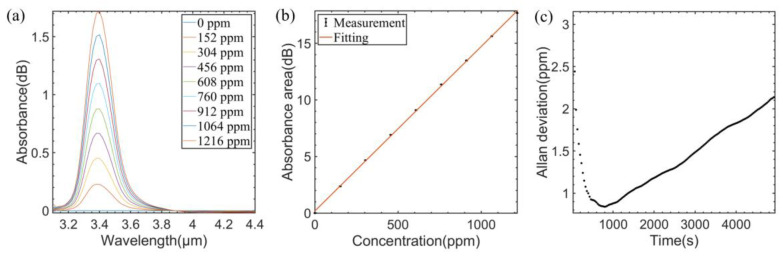
(**a**) Measured absorption spectra of different C_2_H_6_ concentrations. (**b**) Experimental data and fitting curve of C_2_H_6_ concentration versus absorbance area. (**c**) Allan variance analysis of the sensor for C_2_H_6_.

The absorption spectra measured for different concentrations of C_2_H_6_ (with C_2_H_6_ concentration varying from 0 ppm to 1216 ppm, the variation interval was 152 ppm) are shown in Figure 7a. Figure 7b shows the linear fitting between absorbance area and C_2_H_6_ concentrations. The R square value is 0.9996 and the fitting function is expressed as follows:

(9) $$A_{C_{2}H_{6}}=0.0145*c_{C_{2}H_{6}}+0.1800$$

As shown in Figure 7c, the Allan deviation for C_2_H_6_ detection is 926 ppb at an averaging time of 462 s.

The result shows that the low-cost NDIR system, based a commercial infrared light source and a FP detector, achieves ppb-level and ppm-level gas detection. It has excellent gas sensing performance. Therefore, it has the advantages of high accuracy of TDLAS and low cost of NDIR.

### 3.2. Performance of the Sensor with Measured Mixed Gases

**Figure 8:**
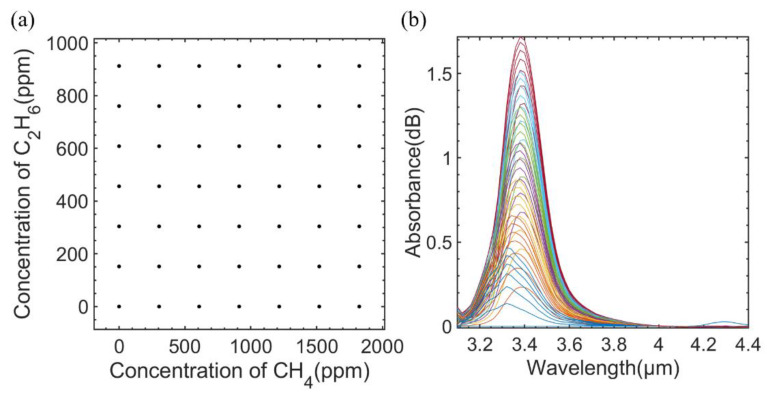
(**a**) Gas concentration distribution of the mixed gases. (**b**) Measured absorption spectra of mixed gases samples.

A PCA-BP neural network algorithm was used to solve the interference of CH_4_ and C_2_H_6_. It needs absorption spectra of mixed gases samples for training. In this paper, the concentration of CH_4_ was set from 0 to 1824 ppm and the concentration of C_2_H_6_ was set from 0 to 912 ppm, respectively. In total, there were 49 different concentration groups of mixed gas samples measured. Figure 8a shows the specific concentration distribution of each mixed sample. The absorption spectra of different concentrations of mixed gases are shown in Figure 8b.

There were 66 spectra data points over the whole wavelength range from 3.1~4.4 µm. Because the absorption band of CO_2_ was from 4.2~4.4 µm, the spectra data range of 3.2~4.0 µm was chosen for the PCA-BP neural network algorithm, which comprised 41 spectral data points. Then, the spectral data points were processed with dimensionality reduction using the PCA algorithm. The contribution rates of the first four principal components are 99.4376%, 0.5598%, 0.0016%, and 0.0003%, respectively, which are more than 99.99% in total. Therefore, the first four principal components are selected to replace the original 41 spectral data points. 

**Figure 9:**
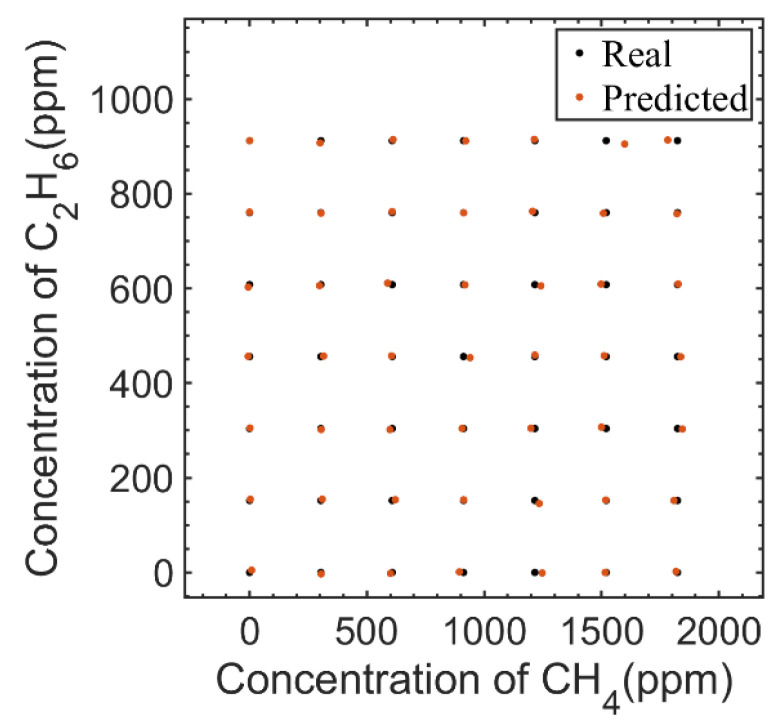
Predicted concentrations of CH_4_ and C_2_H_6_ using measured mixed gases.

After dimension reduction, BP neural network training was carried out. This paper used a three-layer BP neural network. The input was four nodes, that was, four principal component components, and the output was two nodes, that was, the concentrations of CH_4_ and C_2_H_6_. Four hidden layer nodes were selected, the Levenberg–Marquardt algorithm was used for model error training iteration, and the Sigmoid function was used as excitation function. In order to verify the model, leave-one-out cross-validation was used to train and test the 49 groups of measured spectral data, and the validation results are shown in Figure 9. The root mean square error of calibration (RMSEC) and the root mean square error of prediction (RMSEP) were used as the main evaluation indexes of model accuracy for fitting and predicting. The smaller RMSEC value means the higher fitting accuracy of the model, and the smaller RMSEP value means the higher predicted accuracy of the model.

(10) $$\mathrm{RMSEC}=\sqrt{\sum_{i=1}^{n}\frac{{\left(C_{i}-C_{i}^{'}\right)}^{2}}{n}}$$

(11) $$\mathrm{RMSEP}=\sqrt{\sum_{i=1}^{m}\frac{{\left(C_{i}-C_{i}^{'}\right)}^{2}}{m}}$$

where *n* is the number of samples of the training set, *m* is the sample of the verification set, *C_i_* is the real measured concentrations of the samples, and *C_i_*′ is the predicted concentrations of the samples.

The RMSEC of CH_4_ was 1.42 ppm and the RMSEC of C_2_H_6_ was 0.26 ppm. The RMSEP of CH_4_ was 10.97 ppm, and that of C_2_H_6_ was 2.00 ppm. It could be seen that the PCA-BP neural network algorithm can be well applied in this system to solve the problem of CH_4_ and C_2_H_6_ interference.

### 3.3. Simulation-Aid Training

Although using the PCA-BP neural network algorithm could effectively solve the problem of CH_4_ and C_2_H_6_ interference, it needs to measure a large number of samples for training to obtain a great neural network. To improve efficiency, this paper attempted to use a large number of simulation samples for aid training. In addition, the simulation samples are established on a small number of measured samples.

**Figure 10:**
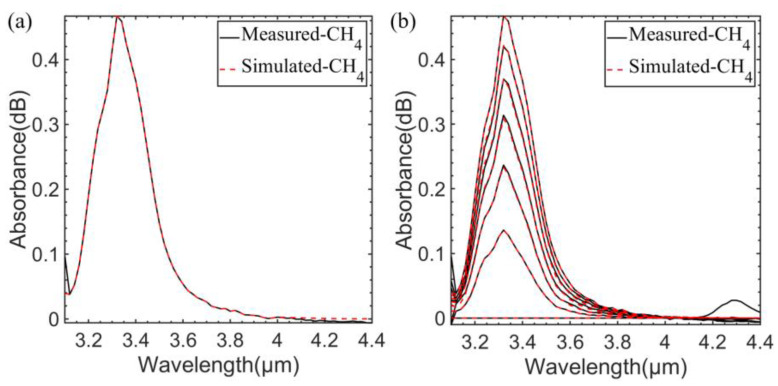
(**a**) Comparison of measured and simulated absorption spectra of 1824 ppm CH_4_. (**b**) Comparison of measured and simulated absorption spectra of CH_4_ at different concentrations.

First, the absorption line intensity and other parameters of CH_4_ and C_2_H_6_ were downloaded from the Hitran database and converted into absorption cross-section data. After the optical path length was determined, the important parameters of simulation, such as window size, fineness, and divergence angle of light source, were inversely deduced from the measured absorption spectra of a known concentration gas. In this paper, the measured absorption spectra of CH_4_ at 1824 ppm were selected as the reference to obtain the simulation parameters. The comparison between the simulated absorption spectra and the measured absorption spectra is shown in Figure 10a. Then, only the CH_4_ concentration was changed to obtain the CH_4_ simulated absorption spectra at different concentrations. The comparison between the simulated absorption spectra and the measured absorption spectra are shown in Figure 10b. The concentration variation range was 0~1824 ppm, and the variation interval was 304 ppm.

**Figure 11:**
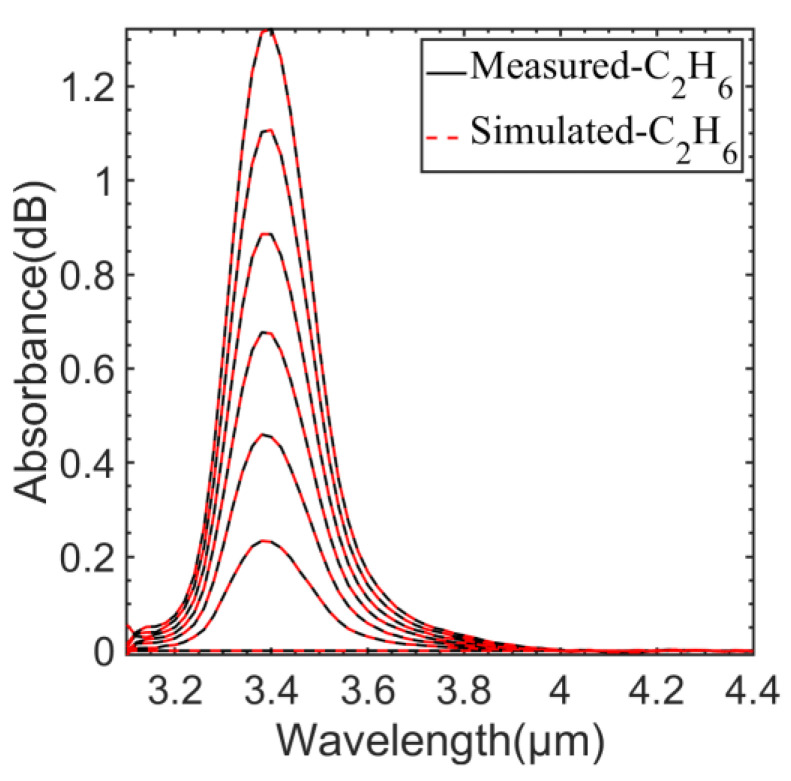
Comparison of measured and simulated absorption spectra of C_2_H_6_ at different concentrations.

Using the same simulated approach, the comparison between the measured absorption spectra and the simulated absorption spectra of C_2_H_6_ are shown in Figure 11. The concentration variation range was 0~912 ppm, and the variation interval was 152 ppm.

Considering the interference of CH_4_ and C_2_H_6_, the mixed simulation absorption spectra data of CH_4_ and C_2_H_6_ cannot be directly obtained from the linear superposition of their single gas absorption spectra. The interference coefficient shall be introduced within the peak interference range of CH_4_ and C_2_H_6_, so the formula for calculating the mixed simulation absorption spectral data was as follows:

(12) $$A\left(\lambda \right)=\{\begin{array}{c} (A_{CH_{4}}\left(\lambda \right)+A_{C_{2}H_{6}}\left(\lambda \right))*\left(1+S\left(\lambda \right)\right),\;3.22<\lambda <3.62\\ A_{CH_{4}}\left(\lambda \right)+A_{C_{2}H_{6}}\left(\lambda \right),\;else \end{array}$$

where *S*(*λ*) was the interference coefficient which could be obtained from several measured mixed absorption spectra and the corresponding CH_4_-C_2_H_6_ superimposed absorption spectra. The value was as follows:

(13) S(λ)=[−0.0094,−0.0129, 0.0010,−0.0008,−0.0069, 0.0025, 0.0128,    0.0122, 0.0125, 0.0106, 0.0091, 0.0048,0.0108,0.0033, 0.0020,−0.0002, 0.0109, 0.0099,−0.0048,−0.0060,−0.0175] 

**Figure 12:**
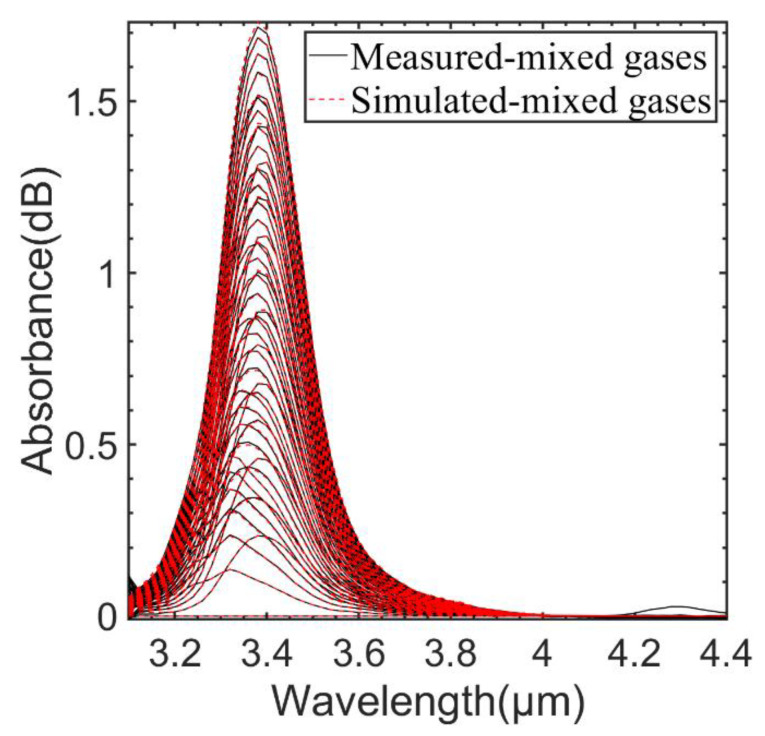
Comparison of measured and simulated absorption spectra of CH_4_ and C_2_H_6_ mixed gases at different concentrations.

We compared the simulated mixed gas absorption spectra obtained according to Equation (12) with the measured mixed gas absorption spectra as shown in Figure 12. The simulated absorption spectra agreed well with the measured absorption spectra. In order to save time, we used simulated absorption spectra to aid in training.

**Figure 13:**
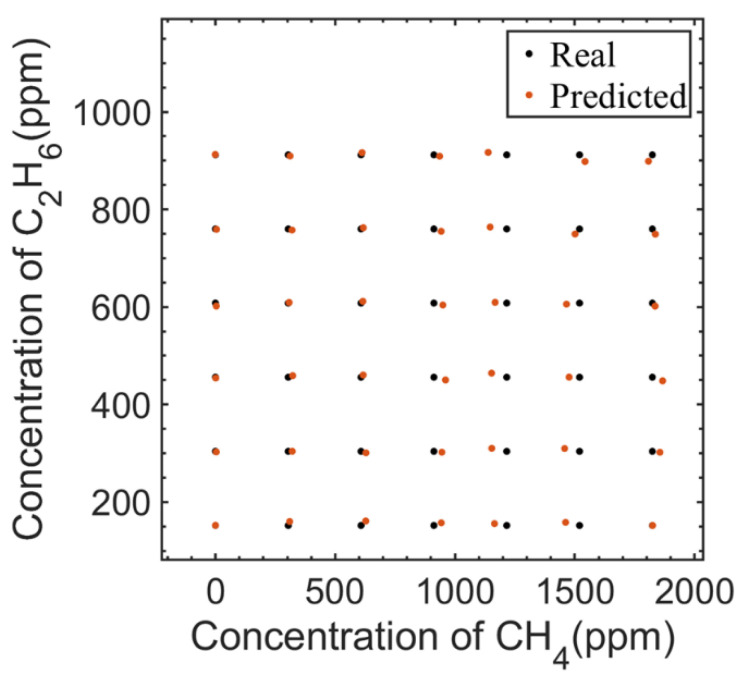
Predicted concentrations of CH_4_ and C_2_H_6_ using simulated mixed gases spectra.

The 49 groups of simulated absorption spectra were used as the training set, and the 49 groups of measured mixed gas absorption spectra data were used as the test set. After dimension reduction by PCA and BP neural network training, the training results were shown in Figure 13. The RMSEP of CH_4_ was 34.99 ppm and the RMSEP of C_2_H_6_ was 3.53 ppm. It can be observed that the RMSEP is larger than that obtained by training with measured spectra data. This is because there is an error between the simulated absorption spectra and the measured absorption spectra. However, using simulated absorption spectra greatly reduced the time cost. Therefore, in some applications that do not require high accuracy, the simulated absorption spectra can be used to replace the measured ones for CH_4_-C_2_H_6_ BP neural network training. The trained neural network can be applied to mixed gas concentration detection, greatly saving time.

**Figure 14:**
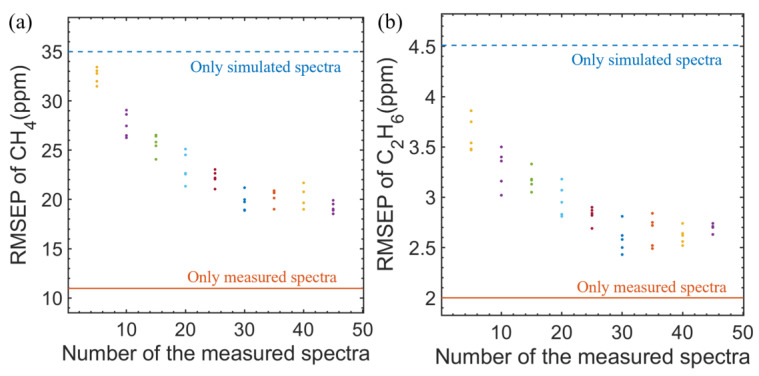
The RMSEP of (**a**) CH_4_ and (**b**) C_2_H_6_ obtained by training with 49 simulated spectra data and different numbers of measured spectra data.

Then, a number of measured absorption spectra data were added into the 49 groups of simulated absorption spectra as the training set. Each training set was trained five times, and the training results are shown in Figure 14a,b. The RMSEP showed a downward trend with the increase in the number of measured spectra data, and between the RMSEP obtained by training with only measured spectra data and only simulated spectra data.

## 4. Conclusions

In this paper, we developed a CH_4_-C_2_H_6_-CO_2_ multi-gas sensing system using a NDIR system. We first studied the detection limit of the system for single gas and found that the detection limit of CO_2_ was 114 ppb at an averaging time of 297 s, that of CH_4_ was 2.59 ppm at an averaging time of 429 s, and that of C_2_H_6_ was 926 ppb at an averaging time of 462 s. Because the absorption spectra of CH_4_ and C_2_H_6_ are highly overlapped, the PCA-BP algorithm is used to obtain the concentrations of CH_4_ and C_2_H_6_ in the mixed gas. The RMSEP of CH_4_ and C_2_H_6_ were 10.97 ppm and 2.00 ppm, respectively. Because the PCA-BP algorithm needs a lot of measured samples for training, it costs a lot of manpower and time. Therefore, this paper proposed a simulation-aid training method, which attempted to use a small number of measured samples to simulate a large number of simulation spectra for aid-training. The RMSEP of CH_4_ and C_2_H_6_ were 34.99 ppm and 3.53 ppm when the simulated spectra data were used for training. 

The gas sensing system proposed in this paper used an infrared broad-spectrum light source and an FP detector, and both of them are commercially available components. HWG served as the transmission medium and gas absorption cell simultaneously. It was directly coupled with the source and detector without any optical components. It greatly improves the stability and portability of the system. Owing to the low-loss property of the HWG, a longer optical path becomes possible, and the performance of the system is improved. The cost of the system is less than 1000 USD. Table 1 summaries the related research using infrared spectroscopy for multi-gas detection. Compared to the TDLAS system, the system in this work has the advantages of a low cost and simple structure. Compared to other NDIR systems, it has a higher accuracy and lower detection error. 

## Figures and Tables

**Table 1:** Summary of related work using infrared absorption spectroscopy to measure CH_4_, C_2_H_6_, and CO_2_.

Method	Target Gas	Detection Limit/Detection Error	Reference	Year
TDLAS	CH_4_	24 ppb	[6]	2020
	CO_2_	144 ppm		
TDLAS	CH_4_	78 ppb	[5]	2022
	C_2_H_6_	190 ppb		
TDLAS	CH_4_	23.53 ppb	[4]	2022
	C_2_H_6_	146.4 ppb		
NDIR	CO	2.96 ppm	[9]	2017
	CO_2_	4.54 ppm		
	CH_4_	2.84 ppm		
NDIR	CH_4_	200 ppm	[28]	2019
	CH_2_O	900 ppm		
	CO_2_	20 ppm		
NDIR	CH_4_	63 ppm	[29]	2020
	CO_2_	2 ppm		
	CO	11 ppm		
NDIR	CO_2_	−0.15%~−0.55%	[7]	2022
	CO	−0.36%~−2.29%		
	C_3_H_8_	2.88%~1.68%		
NDIR	CH_4_	10.97 ppm	Our work	-
	C_2_H_6_	2.00 ppm		
	CO_2_	114 ppb		

## Data Availability

The data that support the findings of this study are available from the corresponding author upon reasonable request.
